# Impact of monocytic differentiation on acute myeloid leukemia patients treated with venetoclax and hypomethylating agents

**DOI:** 10.1002/cam4.7378

**Published:** 2024-07-19

**Authors:** Dian Jin, Jingsong He, Haoguang Chen, Wenjun Wu, Xiaoyan Han, Jing Le, Wenxiu Shu, Qianqian Yang, Shanshan Pei, Zhen Cai, Donghua He

**Affiliations:** ^1^ Bone Marrow Transplantation Center, The First Affiliated Hospital Zhejiang University School of Medicine Hangzhou China; ^2^ Department of Hematology Ningbo Medical Treatment Center Li Huili Hospital Ningbo China; ^3^ Liangzhu Laboratory Zhejiang University Medical Center Hangzhou China; ^4^ Institute of Hematology Zhejiang University Hangzhou China; ^5^ Zhejiang Province Engineering Laboratory for Stem Cell and Immunity Therapy Hangzhou China

**Keywords:** acute myeloid leukemia, cancer risk factor, prognosis, targeted therapy

## Abstract

**Introduction:**

Although the combination of venetoclax (VEN) and hypomethylating agents (HMAs) results in impressive efficacy in acute myeloid leukemia (AML), there is still a subset of patients who are refractory. We investigated the outcomes of AML patients with monocytic differentiation who were treated with frontline VEN/HMA.

**Methods:**

A total of 155 patients with newly diagnosed AML treated with frontline VEN/HMA were enrolled in the study. Monocyte‐like AML was identified by flow cytometry with typical expression of monocytic markers, and M5 was identified according to French, American, and British category. We compared the outcomes of patients with different characteristics.

**Results:**

The rate of complete remission (CR) and CR with incomplete recovery of blood counts (CRi), progression‐free survival (PFS), and overall survival (OS) in monocyte‐like AML were inferior to those in nonmonocyte‐like AML (CR/CRi rates, 26.7% vs. 80.0%, *p* < 0.001; median PFS, 2.1 vs. 8.8 months, *p* < 0.001; median OS, 9.2 vs. 19 months, *p* = 0.013). CR/CRi rate in M5 was lower than that in non‐M5 (60.7% vs. 75.5%, *p* = 0.049). Multivariate analyses showed that monocyte‐like AML was associated with lower odds of CR/CRi and higher risk of progression.

**Conclusion:**

Our study suggested that newly diagnosed AML with a monocytic immunophenotype had a poor prognosis with VEN/HMA treatment.

## Graphical abstract

Several ex vivo studies showed that venetoclax was sensitive to immature blasts but less responsive to mature clones. However, no study described the clinical outcomes of AML with monocytic differentiation in the real world. Our study showed that monocyte‐like AML with high expression of CD4, CD14, CD64, and CD11b and low expression of CD117 had a poor prognosis when treated with frontline VEN/HMA therapy.

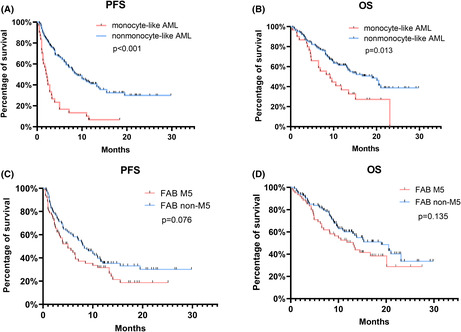

## 1 INTRODUCTION

The BCL‐2 inhibitor venetoclax (VEN) was approved in 2018 by the US Food and Drug Administration and showed excellent efficacy in acute myeloid leukemia (AML). The combination of venetoclax and hypomethylating agents (HMAs) results in impressive response rates of approximately 70% and has emerged as a new standard therapy for older or unfit patients.^1^, ^2^, ^3^ However, there is still a subset of patients who are not sensitive to VEN/HMA therapy. Recently, several studies have sought to identify predictors of response to VEN‐based therapies and have mainly reported that some molecular correlates, such as IDH1/2 mutations and NPM1 mutations, are associated with better outcomes, while TP53 mutations and FLT‐ITD are associated with poorer outcomes.^4^, ^5^, ^6^

On the other hand, the blast maturation state was also suggested to be related to resistance to venetoclax. In ex vivo studies, monocytic subclones with loss of CD117 and upregulation of CD11b, CD68, CD14, and CD64 showed resistance to venetoclax.^7^, ^8^, ^9^ In the real world, however, the impact of cell maturation on the clinical outcome in AML patients treated with VEN/HMA is not very clear. Here, we conducted a retrospective cohort study to explore the association of monocytic differentiation in blast cells and the outcome of newly diagnosed AML patients treated with VEN/HMA therapy.

## 2 METHODS

### 2.1 Patients

All patients with a diagnosis of AML admitted to the First Affiliated Hospital of Zhejiang University School of Medicine and Ningbo Medical Treatment Center Lihuili Hospital from January 2020 to June 2023 were reviewed. The patient eligibility criteria were as follows: (I) ≥18 years of age; (II) newly diagnosed AML; and (III) accepted frontline VEN/HMA treatment for at least 1 cycle and follow‐up to a response assessment or death. The exclusion criteria were as follows: (I) acute promyelocytic leukemia; (II) VEN was treated for less than 7 days per course; (III) received other chemotherapy agents or targeted drugs as a combination; (IV) lack of adequate blast cell immunophenotypic results by flow cytometry; and (V) the treatment strategy was changed when a partial response (PR) was achieved after 1 cycle of VEA/HMA therapy. The study was approved by the Ethical Review Committee of The First Affiliated Hospital of Zhejiang University School of Medicine (approval no. IIT20230704A).

### 2.2 Identification of monocytic AML by FAB category and monocyte‐like AML by immunophenotype

An experienced hematopathologist reviewed the bone marrow smears retrospectively and divided them into M5 (monocytic AML) and non‐M5 by French, American, and British (FAB) category.^10^ Because the FAB classification can be subjective and has been less frequently used in AML, we also used a panel of monocytic phenotypic markers on blast cells tested by flow cytometry at the time of diagnosis to define monocyte‐like AML. Previous studies have shown that monocyte AML usually loses the expression of CD117 and upregulates the expression of the monocytic markers CD4, CD14, CD64, and CD11b.^8^, ^11^, ^12^ Therefore, we developed a scoring model to define monocyte‐like AML and nonmonocyte‐like AML by the five cell surface markers mentioned above: 1 score was given for any positive test of CD4, CD14, CD64, or CD11b and 0.5 score for partial positive test; in contrast, 1 score was given for negative test of CD117 and 0.5 score for partial positivity. Patients with a total score ≥1 were defined as having monocyte‐like AML, and others with a total score <1 were defined as having nonmonocyte‐like AML.

### 2.3 Cytogenetic and molecular data

Baseline cytogenetic testing was available for 149 patients. Diagnostic next‐generation sequencing using a 15‐, 78‐ or 150‐gene targeted panel (including mutations of TP53, IDH1/2, KIT, DNMT3A, and TET2, etc.) was performed in 148 patients. A total of 149 patients were able to be classified into European LeukemiaNet (ELN) 2022 risk group.^13^

### 2.4 Response evaluation and endpoints

Responses were evaluated per modified IWG criteria for AML.^14^ Measurable residual disease (MRD) was assessed by flow cytometry, with negative definition according to ELN guidelines as less than 1000 aberrant blasts.^15^ Progression‐free survival (PFS) was defined as the time from diagnosis to disease progression or death. Overall survival (OS) was defined as the time from the date of diagnosis to death.

### 2.5 Statistical analysis

Chi‐squared test and Wilcoxon Mann–Whitney test were used to compare the baseline characteristics between cohorts. PFS and OS were evaluated by the Kaplan–Meier method with the log‐rank test. Univariable and multivariate logistic regression analyses were conducted to assess the predictive factors for response. Cox regression analyses were conducted to assess the risk factors for PFS. The 95% confidence intervals (CIs) were used to estimate odds ratios (ORs) and hazard ratios (HRs). *p* ≤ 0.05 were considered statistically significant. SPSS v.25 statistical software was used for all analyses, and GraphPad Prism v.9.5.1 was used for graphing.

## 3 RESULTS

### 3.1 Patient characteristics

A total of 155 newly diagnosed AML patients receiving frontline VEN/HMA treatment were enrolled in the study. The median duration of VEN/HMA treatment was 3 (range: 1–10) cycles. The baseline characteristics are listed in Table 1. A total of 76.7% of the monocyte‐like AML patients and 30.4% of the nonmonocyte‐like AML patients were classified as M5 by the FAB classification, with a significant difference (*p* < 0.002). FAB classification, expression of CD4, CD14, CD64, and CD11b, and the total score of monocytic markers for each patient are shown in Table S1. The distribution of ELN risk grades in monocyte‐like AML and nonmonocyte‐like AML were different (*p* = 0.044), but in both monocyte‐like AML and nonmonocyte‐like AML, more than half of patients had ELN adverse disease. In addition, there were more NRAS/KRAS mutations (30.0% vs. 10.7%, *p* = 0.037) and fewer IDH1/2 mutations (16.7% vs. 35.7%, *p* = 0.024) in the monocyte‐like AML group than in the nonmonocyte‐like AML group. The other baseline characteristics in the two groups were similar. The baseline characteristics in the M5 and non‐M5 groups were similar.

**TABLE 1:** Baseline characteristics of patients.

	Monocyte‐like AML (*n* = 30)	Nonmonocyte‐like AML (*n* = 125)	*p*	FAB M5 (*n* = 61)	FAB non‐M5 (*n* = 94)	*p*
Age						
Median (range)	60.5 (24–76)	66 (24–83)	0.074	67 (28–82)	66 (24–83)	0.775
<70	24 (80.0%)	85 (68.0%)	0.196	38 (62.3%)	71 (75.5%)	0.078
≥70	6 (20.0%)	40 (32.0%)		23 (37.7%)	23 (24.5)	
Sex						
Male	14 (46.7%)	66 (52.8%)	0.546	34 (55.7%)	46 (48.9%)	0.408
Female	16 (53.3%)	59 (47.2%)		27 (44.3%)	48 (51.1%)	
Secondary AML	4 (13.3%)	26 (20.8%)	0.353	9 (14.8%)	21 (22.3%)	0.243
Prior HMA	3 (10.0%)	9 (7.2%)	0.893	3 (4.9%)	9 (9.6%)	0.452
Type of HMA						
Azacitidine	26 (86.7%)	109 (89.3%)	0.925	54 (88.5%)	84 (89.4%)	0.875
Decitabine	4 (13.3%)	13 (10.7%)		7 (11.5%)	10 (10.6%)	
Bone marrow blast						
<50%	10 (33.3%)	60 (48.0%)	0.147	26 (42.6%)	44 (46.8%)	0.609
≥50%	20 (66.7%)	65 (52.0%)		35 (57.4%)	50 (53.2%)	
FAB‐M5	23 (76.7%)	38 (30.4%)	<0.001			
ELN 2022 risk group (*n* = 149)						
Favorable	3 (10.3%)	40 (33.3%)	0.044	13 (22.0%)	30 (33.3%)	0.329
Intermediate	7 (24.1%)	18 (15.0%)		11 (18.6%)	14 (15.6%)	
Adverse	19 (65.5%)	62 (51.7%)		35 (59.3%)	46 (51.1%)	
CBF‐AML	0/28 (0.0%)	15/120 (12.5%)	0.104	6/58 (10.3%)	9/90 (10.0%)	0.946
In‐frame bZIP CEBPA mutation	0/28 (0.0%)	13/120 (10.8%)	0.146	3/58 (5.2%)	10/90 (11.1%)	0.213
NPM1 mutation	3/28 (10.7%)	21/120 (17.5%)	0.554	7/58 (12.1%)	12/90 (18.9%)	0.272
FLT3‐ITD mutation	7/28 (25.0%)	17/120 (14.2%)	0.265	11/58 (19.0%)	13/90 (14.4%)	0.466
DNMT3A mutation	11/28 (39.3%)	29/120 (24.2%)	0.105	19/58 (32.8%)	21/90 (23.3%)	0.208
TET2 mutation	2/28 (7.1%)	18/120 (15.0%)	0.4312	5/58 (8.6%)	15/90 (16.7%)	0.162
IDH1/2 mutation	3/28 (10.7%)	36/120 (30.0%)	0.037	13/58 (22.4%)	26/90 (28.9%)	0.383
NRAS/KRAS mutation	10/28 (35.7%)	20/120 (16.7%)	0.024	14/58 (24.1%)	16/90 (17.8%)	0.347
KIT mutation	0/28 (0.0%)	7/120 (5.8%)	0.415	2/58 (3.4%)	5/90 (5.6%)	0.847
TP53 mutation	2/28 (7.1%)	17/120 (14.2%)	0.492	4/58 (6.9%)	15/90 (16.7%)	0.083
Allo‐HSCT	5 (16.7%)	13 (10.4%)	0.519	5 (8.2%)	13 (13.8%)	0.285

Abbreviations: Allo‐HSCT, allogeneic hematopoietic stem cell transplantation; AML, acute myeloid leukemia; CBF, core binding factor; ELN, European LeukemiaNet; FAB, French, American, and British; HMA, hypomethylating agents.

### 3.2 Response, relapse, and early death

**FIGURE 1:**
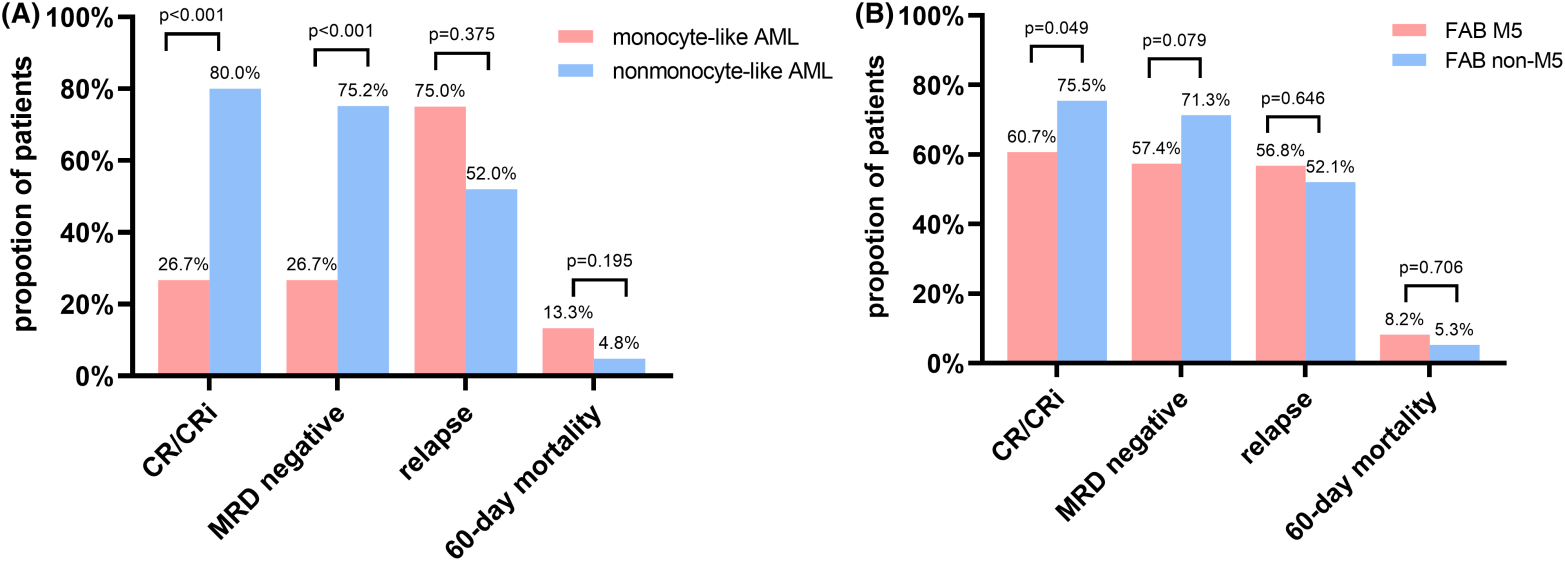
(A) Comparison of response rates, relapse rates, and early deaths in patients with monocyte‐like AML versus nonmonocyte‐like AML. (B) Comparison of response rates, relapse rates, and early deaths in patients with FAB‐M5 versus FAB non‐M5. AML, acute myeloid leukemia; CR, complete remission; CRi, complete remission with incomplete recovery of blood counts; FAB, French, American, and British; MRD, measurable residual disease.

Composite complete remission (complete remission [CR] and CR with incomplete recovery of blood counts [CRi]) for monocyte‐like AML was significantly lower than that for nonmonocyte‐like AML (26.7% vs. 80.0%, *p* < 0.001), as were the MRD‐negative rates (26.7% vs. 75.2%, *p* < 0.001). The CR/CRi rate in M5 was also significantly lower than that in non‐M5 (60.7% vs. 75.5%, *p* = 0.049), while the difference in MRD‐negative rates between the two groups was not significant (57.4% vs. 71.3%, *p* = 075). Relapse rates and 60‐day mortality did not differ between groups (Figure 1).

### 3.3 Survival

**FIGURE 2:**
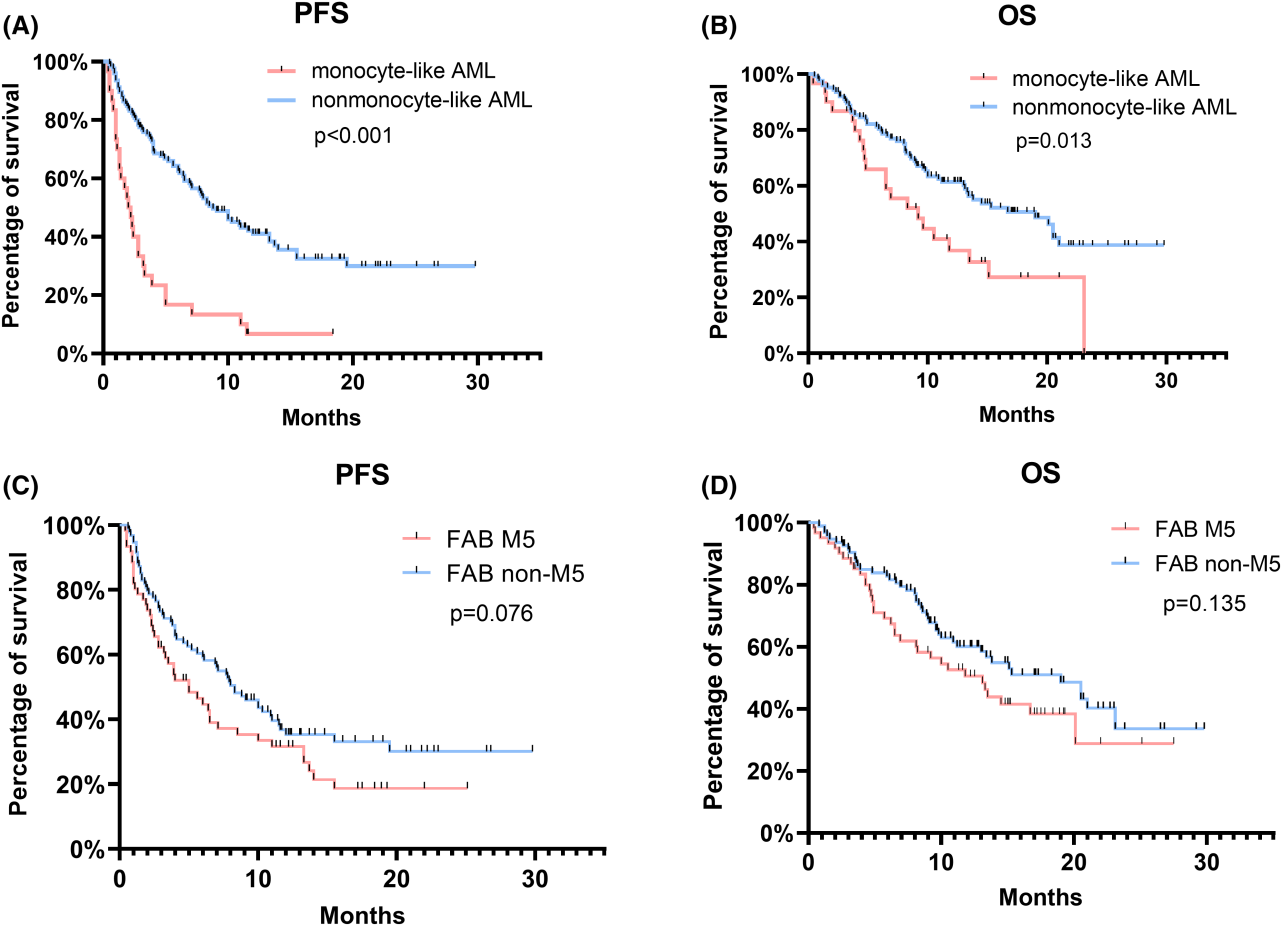
(A) PFS in patients with monocyte‐like AML and nonmonocyte‐like AML. (B) OS in patients with monocyte‐like AML and nonmonocyte‐like AML. (C) PFS in patients with FAB M5 and non‐M5. (D) OS in patients with FAB M5 and non‐M5. AML, acute myeloid leukemia; FAB, French, American, and British; PFS, progression‐free survival; OS, overall survival.

PFS (median 2.1 vs. 8.8 months, *p* < 0.001, Figure 2A) and OS (median 9.2 vs. 19 months, *p* = 0.013, Figure 2B) were significantly shorter in the monocyte‐like AML group than in the nonmonocyte‐like AML group. There was also a trend toward shorter PFS (median 5 vs. 8.3 months, *p* = 0.076) and OS (median 13.1 vs. 19 months, *p* = 0.135) in the M5 group compared to the non‐M5 group, but the differences were not significant (Figure 2C,D).

### 3.4 Subgroup analysis stratified by ELN risk categories

**FIGURE 3:**
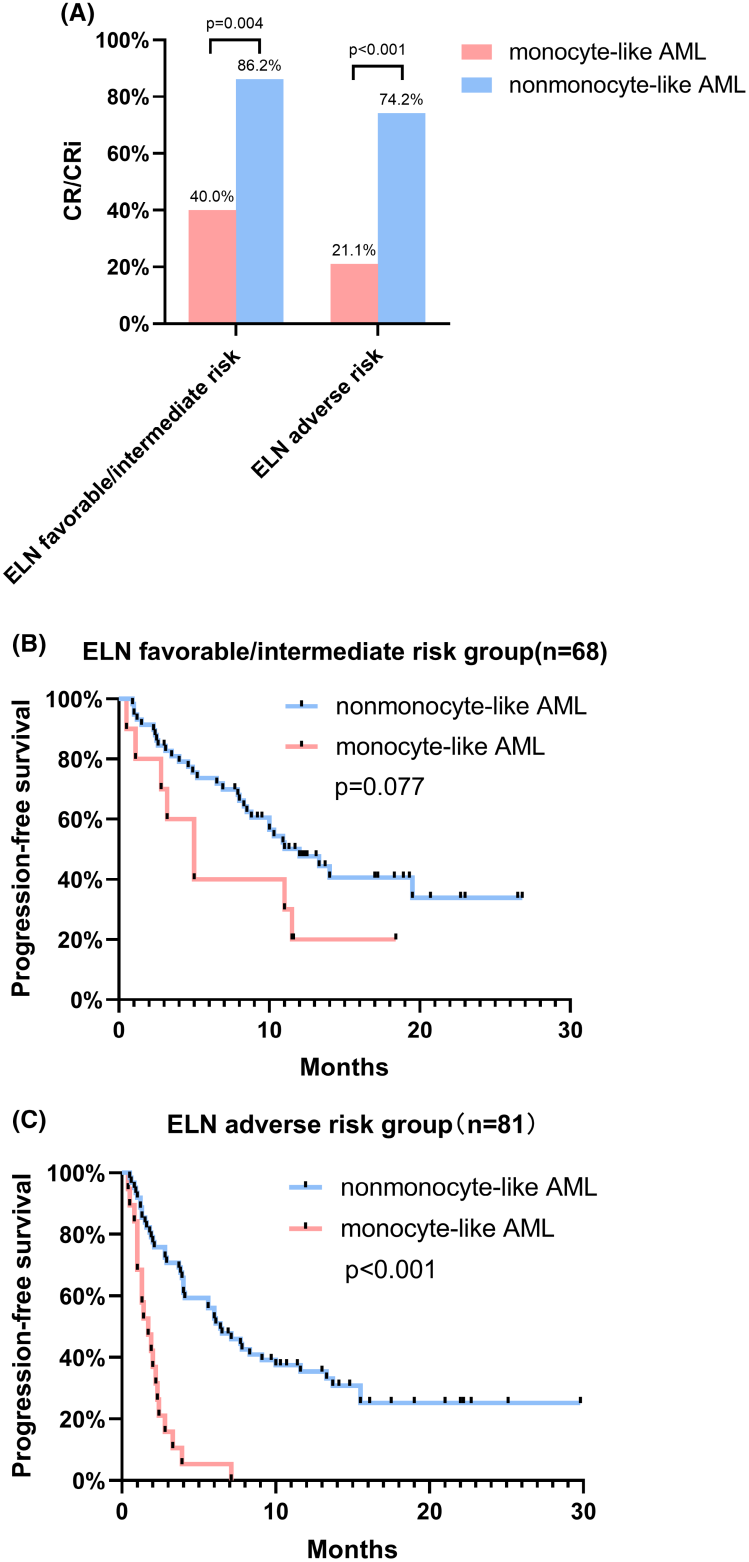
(A) Responses of patients with monocyte‐like AML and nonmonocyte‐like AML in different ELN risk groups. (B) PFS of patients with monocyte‐like AML and nonmonocyte‐like AML in the ELN favorable/intermediate risk group. (C) PFS of patients with monocyte‐like AML and nonmonocyte‐like AML in the ELN adverse risk group. AML, acute myeloid leukemia; CR, complete remission; CRi, complete remission with incomplete recovery of blood counts; ELN, European LeukemiaNet; PFS, progression‐free survival.

We further analyzed the response and PFS of patients with monocyte‐like AML and nonmonocyte‐like AML in the ELN favorable/intermediate risk group and adverse risk group. In both subgroups stratified by ELN risk categories, monocyte‐like AML had lower CR/CRi rates than nonmonocyte‐like AML (Figure 3A). In the ELN favorable/intermediate risk group, the median PFS of patients with monocyte‐like AML and nonmonocyte‐like AML was 5 and 10 months, respectively, with no significant difference (*p* = 0.077, Figure 3B). In the ELN adverse risk group, the median PFS of patients with monocyte‐like AML was significantly shorter than that of patients with nonmonocyte‐like AML (1.7 vs. 6.4 months, *p* < 0.001, Figure 2C).

### 3.5 Multivariate analyses for CR/CRi and progression

Multivariate analyses showed that monocyte‐like AML was associated with lower odds of CR/CRi (OR 0.06, 95% CI 0.02–0.23, *p* < 0.001) and a higher risk of progression (HR 3.05, 95% CI 1.72–5.41, *p* < 0.001). Other factors associated with lower odds of CR/CRi included prior HMA exposure (OR 0.13, 95% CI 0.02–0.68, *p* = 0.016), KIT mutation (OR 0.02, 95% CI 0.00–0.20, *p* = 0.001), and TP53 mutation (OR 0.19, 95% CI 0.05–0.81, *p* = 0.024). Other factors associated with inferior PFS included prior HMA exposure (HR 3.24, 95% CI 1.31–7.97, *p* = 0.011), ELN adverse risk (HR 1.98, 95% CI 1.19–3.29, *p* = 0.008), FLT3‐ITD mutation (HR 1.85, 95% CI 1.07–3.19, *p* = 0.027), and KIT mutation (HR 5.26, 95% CI 2.25–12.28, *p* < 0.001). IDH1/2 mutation (HR 0.40, 95% CI 0.23–0.70, *p* = 0.001) was associated with lower risk of progression (Table 2).

**TABLE 2:** Univariate and multivariate analyses of factors associated with CR/CRi and PFS.

Variables	Logistic regression for CR/CRi				Cox regression for progression			
	Univariate analysis			Multivariate analysis	Univariate analysis		Multivariate analysis	
	OR (95%CI)	*p*	OR (95%CI)	*p*	HR (95%CI)	*p*	HR (95%CI)	*p*
Age ≥ 70	1.57 (0.72–3.45)	0.261			0.97 (0.64–1.47)	0.896		
Male	1.32 (0.66–2.62)	0.430			1.05 (0.71–1.54)	0.810		
Secondary AML	0.84 (0.36–1.97)	0.690			1.53 (0.97–2.42)	0.069	0.98 (0.51–1.87)	0.938
Prior HMA	0.28 (0.03–0.93)	0.037	0.13 (0.02–0.68)	0.016	2.23 (1.19–4.19)	0.012	3.24 (1.31–7.97)	0.011
Decitabine	1.05 (0.35–3.17)	0.931			0.84 (0.44–1.62)	0.610		
Bone marrow blast ≥ 50%	0.86 (0.43–1.72)	0.667			1.04 (0.71–1.53)	0.845		
Monocyte‐like AML	0.09 (0.04–0.23)	<0.001	0.06 (0.02–0.23)	<0.001	3.31 (2.12–5.16)	<0.001	3.05 (1.72–5.41)	<0.001
FAB‐M5	0.50 (0.25–1.00)	0.051	0.57 (0.19–1.75)	0.324	1.42 (0.96–2.09)	0.079	1.14 (0.71–1.82)	0.591
ELN adverse risk	0.42 (0.20–0.88)	0.021	0.52 (0.15–1.833)	0.306	1.83 (1.23–2.74)	0.003	1.98 (1.19–3.29)	0.008
CBF‐AML	0.86 (0.28–2.68)	0.795			0.68 (0.22–1.41)	0.303		
In‐frame bZIP CEBPA mutation	NA	0.999			0.69 (0.33–1.42)	0.311		
NPM1 mutation	5.8 (1.31–26.02)	0.021	2.70 (0.42–17.51)	0.297	0.53 (0.29–0.97)	0.039	0.75 (0.37–1.51)	0.420
FLT3‐ITD	0.45 (0.18–1.09)	0.077	0.36 (0.09–1.43)	0.145	1.85 (1.11–3.06)	0.018	1.85 (1.07–3.19)	0.027
DNMT3A	1.72 (0.74–4.00)	0.206			0.83 (0.53–1.30)	0.415		
TET2	1.89 (0.59–5.99)	0.283			1.00 (0.58–1.73)	0.994		
IDH1/2 mutation	3.94 (1.43–10.89)	0.008	2.48 (0.71–8.62)	0.153	0.43 (0.26–0.71)	0.001	0.40 (0.23–0.70)	0.001
NRAS/KRAS mutation	0.49 (0.21–1.11)	0.088	0.62 (0.19–2.08)	0.439	1.11 (0.68–1.81)	0.688		
KIT mutation	0.06 (0.01–0.55)	0.012	0.02 (0.00–0.20)	0.001	3.64 (1.67–7.97)	0.001	5.26 (2.25–12.28)	<0.001
TP53 mutation	0.34 (0.13–0.89)	0.029	0.19 (0.05–0.81)	0.024	1.69 (0.97–2.93)	0.063	1.57 (0.85–2.91)	0.152
Allo‐HSCT	1.60 (0.50–5.15)	0.430			0.63 (0.33–1.22)	0.172		

Abbreviations: Allo‐HSCT, allogeneic hematopoietic stem cell transplantation; AML, acute myeloid leukemia; CBF, core binding factor; CI, confidence interval; CR, complete remission; CRi, complete remission with incomplete recovery of blood counts; ELN, European LeukemiaNet; FAB, French, American, and British; HR, hazard ratio; OR, odds ratio; PFS, progression‐free survival.

## 4 DISCUSSION

The outcomes of patients treated with VEN/HMA are different, and it is urgent to identify factors that predict prognosis. Several ex vivo studies have shown that venetoclax was sensitive to immature blasts with high expression of primitive maturation markers (CD34 and CD117) but less responsive to mature clones with high expression of monocytic markers (CD14, CD11b, CD64, and CD68).^7^, ^8^, ^9^, ^16^, ^17^, ^18^ However, there are very few studies describing the clinical outcomes of AML with monocytic differentiation in the real world. Pollyea et al. conducted a multivariate analysis and showed that FAB‐M5 favored intensive chemotherapy over venetoclax/azacitidine for CR/CRi.^4^ Pei et al. reported a retrospective study of 100 AML patients treated with venetoclax/azacitidine, and multivariate analysis revealed that FAB‐M5 was a predictor of refractory disease.^8^ However, FAB classification can be highly subjective. In this study, we added a new classification of monocyte‐like AML based on the scoring of cell surface markers on blast cells to investigate the treatment response and survival of newly diagnosed AML with monocytic differentiation treated with VEN/HMA.

Our study showed that monocyte‐like AML with high expression of CD4, CD14, CD64, and CD11b and low expression of CD117 had a poor prognosis when treated with frontline VEN/HMA therapy. The response rate, PFS, and OS in monocyte‐like AML were all much inferior to those in nonmonocyte‐like AML. When grouped by FAB category, patients with M5 had a lower response rate than non‐M5 patients, but the differences in PFS and OS were not significant. Moreover, in multivariate analysis, monocyte‐like AML also showed independent effects on poor response and survival, whereas M5 did not. These results suggested that the monocytic immunophenotype of tumor cells by flow cytometry may be a better indicator of poor response to VEN/HMA therapy than FAB classification. In addition, after stratification by ELN risk, monocyte‐like AML still had a worse prognosis than nonmonocyte‐like AML in each subgroup, especially in the adverse‐risk group. Therefore, we suggest that when determining induction therapy for AML, the expression of monocytic AML surface markers can be used as important evidence for the selection of the VEN/HMA regimen.

The mechanism of resistance to venetolcax in monocytic AML is reported to be caused by loss of BCL2 dependence during monocytic differentiation.^8^ Data suggest that BCL2 loss is a conserved evolutionary feature during both normal and malignant monocytic development; however, the exact mechanism of this evolutionary selection is not understood.^8^, ^19^, ^20^ One possible explanation comes from the perspective of energy metabolism. Studies found that monocytic AML preferentially relies on MCL1 rather than BCL2 for their mode of energy metabolism.^8^, ^21^, ^22^

In multivariate analysis, ELN adverse risk was associated with inferior prognosis, which was consistent with data in the pre‐venetoclax era. In patients treated with venetoclax‐based therapies, TP53 mutations and FLT3‐ITD mutations were reported to be with adverse outcome, while IDH1/2 mutations were with better outcome.^4^, ^5^, ^6^ The results of our study were similar to those reported above, addressing the importance of genetic characteristics on the efficacy of VEN/HMA therapies.

Interestingly in our study, we found that the KIT mutation was an independent risk factor for inferior prognosis in newly diagnosed AML patients treated with VEN/HMA, which has not been previously reported. KIT mutations have been found in approximately 4%–6% of newly diagnosed adult AML and 20%–40% of CBF‐AML.^23^, ^24^, ^25^, ^26^ In some studies, KIT mutations were associated with poor prognosis in CBF‐AML, particularly with t (8;21).^27^, ^28^, ^29^, ^30^ However, others suggested that MRD may be a more relevant prognostic factor to KIT mutations in CBF‐AML.^31^, ^32^, ^33^ Based on these data, the latest NCCN and ELN guidelines do not consider KIT mutations as a factor for risk stratifications.^13^, ^34^ However, the clinical outcomes of patients with KIT mutations receiving venetoclax‐based therapy have not been reported in the literature, nor has the possible mechanism of KIT mutations and venetoclax resistance. In this study, we first reported that KIT mutation predicted a lower response rate and worse PFS in newly diagnosed AML patients treated with VEN/HMA, and it was even more relevant to prognosis than ELN risk stratification. However, only seven patients in our study have KIT mutation, which may lead to bias in analysis. The effect of KIT mutation on the prognosis of newly diagnosed AML treated with VEN/HMA needs to be further confirmed by studies with larger sample sizes.

Finally, we would like to note a few limitations of the current study. First, because it was a retrospective study, the baseline characteristics with respect to ELN risk stratification, IDH1/2 mutations, and NRAS/KRAS mutations between groups differed, and the dosages of venetoclax varied. Second, the treatment strategies after achieving remission differed, which could lead to bias in PFS and OS. Lastly, the small sample sizes limited detailed analysis in different subgroups.

## 5 CONCLUSIONS

Our study suggested that newly diagnosed monocyte‐like AML with high expression of CD4, CD14, CD64, and CD11b and low expression of CD117 had a poor response to frontline VEN/HMA treatment. When determining induction therapy for AML, we should carefully evaluate the expression of monocytic markers and their potential resistance to venetoclax‐based therapies, especially in adverse risk groups.

## AUTHOR CONTRIBUTIONS

**Dian Jin:** Formal analysis (equal); methodology (equal); writing – original draft (equal). **Jingsong He:** Formal analysis (equal); methodology (equal); writing – original draft (equal). **Haoguang Chen:** Formal analysis (equal); methodology (equal); writing – original draft (equal). **Wenjun Wu:** Data curation (equal); writing – review and editing (equal). **Xiaoyan Han:** Investigation (equal); resources (equal). **Jing Le:** Investigation (equal); resources (equal). **Wenxiu Shu:** Investigation (equal); resources (equal). **Qianqian Yang:** Investigation (equal); resources (equal). **Shanshan Pei:** Conceptualization (equal); funding acquisition (equal). **Zhen Cai:** Conceptualization (equal); funding acquisition (equal). **Donghua He:** Conceptualization (equal); funding acquisition (equal).

## FUNDING INFORMATION

This work was supported by National Natural Science Foundation of China (81770217, 81872322, and 82270157), Natural Science Foundation of Zhejiang Province (LY22H080003 and LQ22H080008), and Zhejiang Key Research and Development Project (2020C03014).

## CONFLICT OF INTEREST STATEMENT

The authors have no conflict of interest to delcare.

## ETHICS STATEMENT

*Approval of the research protocol by an Institutional Reviewer Board*: The study was approved by the Ethical Review Committee of the First Affiliated Hospital of Zhejiang University School of Medicine (approval no. IIT20230704A). All methods used in this study follow the principles of the Declaration of Helsinki. As this is a retrospective study, the requirement for written informed consent was waived. *Informed consent*: N/A. Registry and the *registration no. of the study/trial*: N/A. *Animal studies*: N/A.

## Supporting information


Table S1.

## Data Availability

The data that support the findings of this study are available from the corresponding author upon reasonable request.
