# Supplementary material for: Impact of monocytic differentiation on acute myeloid leukemia patients treated with venetoclax and hypomethylating agents
Source: Cancer Med. 2024 Jul 19;13(14):e7378. doi: 10.1002/cam4.7378 (PMC11258555; doi:10.1002/cam4.7378)
Supplement: Supplementary file 1 — Table S1. [file CAM4-13-e7378-s001.docx]

| Table S1. FAB classification, expression of CD4, CD14, CD64, and CD11b, and the total score of monocytic markers for each patient. | | | | | | | |
| --- | --- | --- | --- | --- | --- | --- | --- |
| patient ID | CD117 | CD4 | CD14 | CD64 | CD11b | total score | FAB |
| 1 | positive | negative | negative | negative | negative | 0 | M2 |
| 2 | positive | negative | positive | negative | positive | 2 | M5 |
| 3 | positive | negative | negative | negative | negative | 0 | M2 |
| 4 | positive | negative | positive | positive | positive | 3 | M5 |
| 5 | positive | partial positive | partial positive | partial positive | negative | 1.5 | M5 |
| 6 | positive | negative | negative | negative | negative | 0 | M1 |
| 7 | negative | negative | positive | positive | positive | 4 | M5 |
| 8 | positive | negative | negative | negative | negative | 0 | M2 |
| 9 | positive | partial positive | partial positive | partial positive | partial positive | 2 | M5 |
| 10 | positive | negative | negative | partial positive | negative | 0.5 | M5 |
| 11 | positive | negative | negative | negative | negative | 0 | M5 |
| 12 | positive | negative | negative | negative | negative | 0 | M2 |
| 13 | positive | negative | negative | negative | negative | 0 | M2 |
| 14 | positive | negative | negative | negative | partial positive | 0.5 | M2 |
| 15 | positive | negative | negative | negative | negative | 0 | M2 |
| 16 | positive | negative | negative | negative | negative | 0 | M5 |
| 17 | positive | negative | negative | negative | negative | 0 | M2 |
| 18 | positive | negative | negative | negative | partial positive | 0.5 | M5 |
| 19 | positive | negative | positive | positive | partial positive | 2.5 | M5 |
| 20 | positive | negative | negative | negative | negative | 0 | M5 |
| 21 | positive | negative | negative | negative | negative | 0 | M2 |
| 22 | positive | negative | negative | negative | negative | 0 | M2 |
| 23 | positive | negative | negative | negative | negative | 0 | M2 |
| 24 | positive | negative | negative | partial positive | negative | 0.5 | M5 |
| 25 | positive | negative | negative | negative | negative | 0 | M0 |
| 26 | positive | negative | negative | partial positive | negative | 0.5 | M2 |
| 27 | positive | negative | negative | negative | negative | 0 | M5 |
| 28 | positive | negative | negative | negative | negative | 0 | M2 |
| 29 | positive | negative | negative | negative | negative | 0 | M2 |
| 30 | positive | negative | negative | negative | negative | 0 | M2 |
| 31 | positive | negative | negative | negative | negative | 0 | M2 |
| 32 | negative | partial positive | positive | positive | positive | 4.5 | M5 |
| 33 | positive | negative | negative | partial positive | partial positive | 1 | M5 |
| 34 | positive | negative | negative | negative | partial positive | 0.5 | M2 |
| 35 | positive | negative | negative | negative | negative | 0 | M5 |
| 36 | positive | negative | negative | negative | partial positive | 0.5 | M2 |
| 37 | positive | negative | negative | negative | negative | 0 | M2 |
| 38 | partial positive | negative | negative | negative | negative | 0.5 | M5 |
| 39 | positive | negative | negative | partial positive | negative | 0.5 | M1 |
| 40 | positive | negative | negative | negative | negative | 0 | M2 |
| 41 | positive | negative | negative | negative | negative | 0 | M0 |
| 42 | positive | negative | negative | negative | negative | 0 | M2 |
| 43 | positive | negative | negative | negative | negative | 0 | M2 |
| 44 | positive | negative | negative | partial positive | negative | 0.5 | M5 |
| 45 | positive | negative | negative | partial positive | negative | 0.5 | M2 |
| 46 | positive | negative | negative | negative | negative | 0 | M0 |
| 47 | positive | negative | negative | negative | negative | 0 | M2 |
| 48 | positive | negative | negative | negative | negative | 0 | M2 |
| 49 | positive | negative | negative | negative | negative | 0 | M2 |
| 50 | positive | negative | negative | negative | negative | 0 | M2 |
| 51 | positive | negative | negative | negative | negative | 0 | M2 |
| 52 | positive | negative | negative | negative | negative | 0 | M0 |
| 53 | positive | negative | negative | negative | negative | 0 | M2 |
| 54 | positive | negative | negative | partial positive | negative | 0.5 | M0 |
| 55 | positive | negative | negative | partial positive | negative | 0.5 | M5 |
| 56 | positive | negative | negative | negative | negative | 0 | M5 |
| 57 | positive | negative | negative | negative | negative | 0 | M2 |
| 58 | positive | negative | negative | partial positive | negative | 0.5 | M5 |
| 59 | positive | negative | negative | negative | negative | 0 | M5 |
| 60 | positive | negative | negative | partial positive | negative | 0.5 | M2 |
| 61 | positive | negative | negative | negative | negative | 0 | M5 |
| 62 | positive | negative | negative | negative | negative | 0 | M5 |
| 63 | positive | negative | negative | partial positive | partial positive | 1 | M2 |
| 64 | positive | negative | negative | negative | negative | 0 | M5 |
| 65 | positive | negative | negative | negative | negative | 0 | M2 |
| 66 | positive | negative | negative | partial positive | negative | 0.5 | M5 |
| 67 | partial positive | negative | partial positive | positive | partial positive | 2.5 | M5 |
| 68 | positive | negative | negative | negative | negative | 0 | M2 |
| 69 | positive | negative | negative | negative | negative | 0 | M2 |
| 70 | positive | negative | negative | negative | negative | 0 | M2 |
| 71 | partial positive | negative | negative | partial positive | negative | 1 | M2 |
| 72 | positive | negative | negative | partial positive | negative | 0.5 | M2 |
| 73 | positive | negative | negative | negative | negative | 0 | M2 |
| 74 | positive | partial positive | partial positive | partial positive | partial positive | 2 | M5 |
| 75 | positive | negative | negative | partial positive | partial positive | 1 | M2 |
| 76 | positive | negative | negative | negative | negative | 0 | M2 |
| 77 | positive | negative | negative | negative | negative | 0 | M5 |
| 78 | positive | negative | negative | negative | negative | 0 | M5 |
| 79 | positive | negative | negative | negative | negative | 0 | M2 |
| 80 | positive | negative | negative | negative | negative | 0 | M5 |
| 81 | positive | negative | negative | positive | negative | 1 | M5 |
| 82 | positive | negative | negative | positive | negative | 1 | M5 |
| 83 | positive | negative | negative | partial positive | negative | 0.5 | M2 |
| 84 | positive | negative | negative | positive | negative | 1 | M2 |
| 85 | positive | negative | negative | negative | negative | 0 | M2 |
| 86 | positive | negative | negative | negative | negative | 0 | M5 |
| 87 | positive | negative | negative | negative | negative | 0 | M1 |
| 88 | positive | negative | negative | partial positive | negative | 0.5 | M5 |
| 89 | positive | negative | negative | partial positive | negative | 0.5 | M2 |
| 90 | positive | negative | negative | negative | negative | 0 | M2 |
| 91 | positive | negative | negative | partial positive | negative | 0.5 | M5 |
| 92 | positive | negative | negative | negative | negative | 0 | M2 |
| 93 | negative | positive | negative | positive | positive | 4 | M5 |
| 94 | partial positive | partial positive | partial positive | partial positive | partial positive | 2.5 | M2 |
| 95 | positive | negative | negative | negative | negative | 0 | M2 |
| 96 | positive | negative | negative | negative | partial positive | 0.5 | M5 |
| 97 | positive | negative | negative | partial positive | partial positive | 1 | M0 |
| 98 | positive | negative | negative | negative | negative | 0 | M1 |
| 99 | positive | negative | negative | negative | partial positive | 0.5 | M2 |
| 100 | positive | negative | negative | negative | negative | 0 | M2 |
| 101 | positive | negative | negative | negative | negative | 0 | M5 |
| 102 | negative | negative | partial positive | positive | partial positive | 3 | M5 |
| 103 | positive | negative | negative | negative | partial positive | 0.5 | M5 |
| 104 | negative | positive | positive | positive | positive | 5 | M5 |
| 105 | positive | negative | negative | negative | negative | 0 | M2 |
| 106 | positive | negative | negative | negative | negative | 0 | M5 |
| 107 | positive | negative | negative | partial positive | negative | 0.5 | M2 |
| 108 | positive | negative | negative | partial positive | negative | 0.5 | M5 |
| 109 | positive | negative | negative | partial positive | negative | 0.5 | M2 |
| 110 | positive | partial positive | negative | partial positive | negative | 1 | M5 |
| 111 | positive | negative | negative | negative | negative | 0 | M2 |
| 112 | partial positive | negative | negative | negative | positive | 1.5 | M5 |
| 113 | positive | negative | partial positive | partial positive | partial positive | 1.5 | M5 |
| 114 | positive | negative | negative | negative | negative | 0 | M2 |
| 115 | positive | negative | negative | negative | negative | 0 | M5 |
| 116 | negative | negative | partial positive | positive | partial positive | 3 | M5 |
| 117 | positive | negative | negative | negative | negative | 0 | M2 |
| 118 | positive | negative | negative | positive | partial positive | 1.5 | M5 |
| 119 | positive | negative | negative | negative | negative | 0 | M0 |
| 120 | positive | negative | negative | partial positive | negative | 0.5 | M2 |
| 121 | positive | negative | negative | partial positive | negative | 0.5 | M2 |
| 122 | positive | negative | negative | partial positive | negative | 0.5 | M5 |
| 123 | positive | negative | negative | negative | negative | 0 | M2 |
| 124 | positive | negative | negative | negative | negative | 0 | M2 |
| 125 | positive | negative | negative | negative | negative | 0 | M4 |
| 126 | positive | negative | negative | partial positive | negative | 0.5 | M4 |
| 127 | positive | negative | negative | negative | negative | 0 | M0 |
| 128 | positive | negative | negative | negative | negative | 0 | M2 |
| 129 | positive | negative | negative | partial positive | negative | 0.5 | M2 |
| 130 | positive | negative | negative | negative | negative | 0 | M5 |
| 131 | positive | negative | negative | partial positive | negative | 0.5 | M2 |
| 132 | positive | negative | negative | negative | negative | 0 | M5 |
| 133 | positive | negative | negative | partial positive | negative | 0.5 | M5 |
| 134 | positive | negative | negative | partial positive | negative | 0.5 | M2 |
| 135 | positive | negative | negative | negative | negative | 0 | M2 |
| 136 | positive | negative | negative | negative | negative | 0 | M5 |
| 137 | positive | negative | negative | negative | negative | 0 | M2 |
| 138 | positive | negative | negative | partial positive | negative | 0.5 | M5 |
| 139 | positive | negative | negative | partial positive | partial positive | 1 | M5 |
| 140 | partial positive | negative | negative | positive | negative | 1.5 | M4 |
| 141 | negative | negative | negative | positive | partial positive | 2.5 | M5 |
| 142 | positive | negative | negative | negative | negative | 0 | M2 |
| 143 | positive | negative | negative | negative | negative | 0 | M5 |
| 144 | positive | negative | negative | negative | negative | 0 | M2 |
| 145 | positive | negative | negative | negative | negative | 0 | M2 |
| 146 | positive | negative | negative | partial positive | negative | 0.5 | M2 |
| 147 | positive | negative | negative | negative | negative | 0 | M2 |
| 148 | positive | negative | negative | negative | negative | 0 | M0 |
| 149 | positive | negative | negative | negative | negative | 0 | M2 |
| 150 | positive | negative | negative | negative | negative | 0 | M2 |
| 151 | partial positive | negative | negative | positive | negative | 1.5 | M5 |
| 152 | positive | negative | negative | partial positive | negative | 0.5 | M2 |
| 153 | positive | negative | negative | partial positive | negative | 0.5 | M2 |
| 154 | positive | negative | negative | partial positive | negative | 0.5 | M2 |
| 155 | positive | negative | negative | negative | negative | 0 | M5 |
| Abbreviations: FAB: French, American, and British. | | | | | | | |
